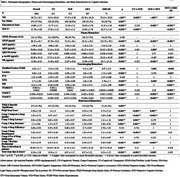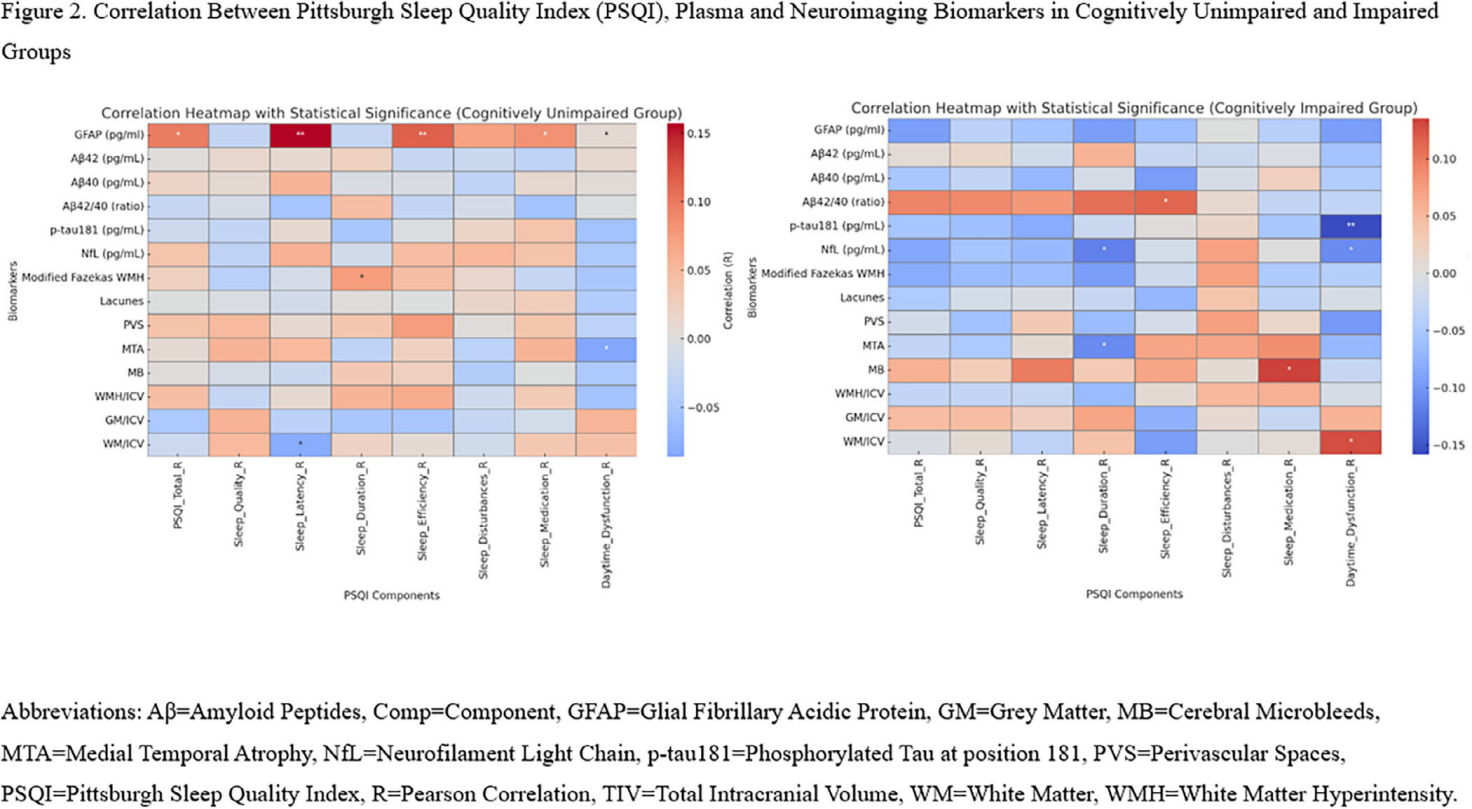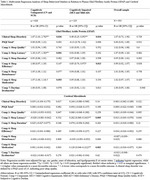# Rest Interrupted: Plasma GFAP, Cerebral Microbleeds, and Sleep Disturbances in Neurodegeneration – Insights from the Southeast Asian BIOCIS Study

**DOI:** 10.1002/alz70857_102597

**Published:** 2025-12-25

**Authors:** Yi Jin Leow, Justin Jit Hong Ong, Jia Dong James Wang, Nagaendran Kandiah

**Affiliations:** ^1^ Lee Kong Chian School of Medicine, Singapore, Singapore, Singapore; ^2^ Lee Kong Chian School of Medicine, Nanyang Technological University, Singapore, Singapore; ^3^ Lee Kong Chian School of Medicine, Nanyang Technological University, Singapore, Singapore, Singapore

## Abstract

**Background:**

Rest is the cornerstone of mental clarity. Chronic sleep disturbances disrupt these processes, driving neuroinflammation, astrocytic dysfunction and neurodegeneration. Glial fibrillary acidic protein (GFAP), a marker of astrocytic activation, has been linked to sleep‐wake disruptions in Alzheimer's disease (AD). Cerebral microbleeds (CMBs), reflecting cerebral small vessel disease (CSVD) also contribute to sleep disturbances. However, the interplay between GFAP, CMBs, and sleep disturbances across cognitive impairment stages remains underexplored.

**Methods:**

This cross‐sectional study included 1,801 community‐dwelling participants in Singapore from the Biomarkers and Cognition Study, Singapore (mean age:58.7±10.7years; 37.4%male). Participants were categorized as cognitively unimpaired (CU: cognitively normal or subjective cognitive decline) or cognitively impaired (CI: mild cognitive impairment[MCI] or mild AD) based on standardized diagnostic criteria. Plasma GFAP, APOE ε4 status, and sleep quality were assessed. Brain imaging was conducted with a 3T Siemens Prisma Fit MRI scanner, and CSVD markers were visually graded using validated scales. ANOVA, correlation, and multivariate regression analyses examined associations between GFAP, CMBs, and sleep disturbances in the overall sample, CU, and CI groups.

**Results:**

In the CU group, higher plasma GFAP levels were significantly associated with increased odds of poor sleep (OR=1.67, 95% CI[1.03, 2.70], *p* = .036) and longer sleep latency (OR=1.18, 95% CI[1.07, 1.30], *p* = .001). Conversely, in the CI group, higher GFAP levels were significantly associated with reduced odds of poor sleep (OR=0.49, 95% CI[0.25, 0.95], *p* = .034) and higher sleep efficiency (OR=0.85, 95% CI[0.74, 0.97], *p* = .013).

CMBs were significantly associated with specific PSQI subcomponents in the CI group, including longer sleep latency (OR=0.202, 95% CI[0.060, 0.343], *p* = .005) and increased use of sleep medication (OR=0.129, 95% CI[0.046, 0.212], *p* = .002).

**Conclusion:**

This study highlights the dual contributions of neuroinflammatory (i.e., GFAP) and cerebrovascular (i.e., CMBs) in sleep disturbances across cognitive stages. In CU individuals, elevated GFAP likely reflects astrocytic activation disrupting sleep homeostasis. In CI individuals, adaptive astrocytic responses or diminished subjective awareness may explain improved sleep outcomes. CMBs exacerbate specific sleep disturbances in CI individuals, highlighting vascular contributions to sleep regulation. Addressing sleep disturbances could mitigate neurodegeneration, highlighting the need for targeted interventions and public health strategies. Future studies integrating biological sleep assessments is essential to elucidate the interplay between sleep, GFAP, and cerebrovascular pathology.